# e!DAL - a framework to store, share and publish research data

**DOI:** 10.1186/1471-2105-15-214

**Published:** 2014-06-24

**Authors:** Daniel Arend, Matthias Lange, Jinbo Chen, Christian Colmsee, Steffen Flemming, Denny Hecht, Uwe Scholz

**Affiliations:** 1 Leibniz Institute of Plant Genetics and Crop Plant Research (IPK), OT Gatersleben, Corrensstr. 3, 06466 Stadt Seeland, Germany

**Keywords:** Research data management, Data publication, Persistent identifier, Metadata annotation, Shared repositories, JAVA API

## Abstract

**Background:**

The life-science community faces a major challenge in handling “big data”, highlighting the need for high quality infrastructures capable of sharing and publishing research data. Data preservation, analysis, and publication are the three pillars in the “big data life cycle”. The infrastructures currently available for managing and publishing data are often designed to meet domain-specific or project-specific requirements, resulting in the repeated development of proprietary solutions and lower quality data publication and preservation overall.

**Results:**

*e!DAL* is a lightweight software framework for publishing and sharing research data. Its main features are version tracking, metadata management, information retrieval, registration of persistent identifiers (DOI), an embedded HTTP(S) server for public data access, access as a network file system, and a scalable storage backend. *e!DAL* is available as an API for local non-shared storage and as a remote API featuring distributed applications. It can be deployed “out-of-the-box” as an on-site repository.

**Conclusions:**

*e!DAL* was developed based on experiences coming from decades of research data management at the Leibniz Institute of Plant Genetics and Crop Plant Research (IPK). Initially developed as a data publication and documentation infrastructure for the IPK’s role as a data center in the DataCite consortium, *e!DAL* has grown towards being a general data archiving and publication infrastructure. The *e!DAL* software has been deployed into the Maven Central Repository. Documentation and Software are also available at: http://edal.ipk-gatersleben.de.

## Background

The availability of cross-domain data has increased dramatically over the last decade, driven by forces including systems biology, imaging for phenomics, and high-throughput technologies such as next-generation sequencing (NGS). As a consequence, the life sciences have become one of the most data-intensive sciences and a major player in the “big data” and “e-science” age [1,2]. Roos remarked over 10 years ago that “we are swimming in a rapidly rising sea of data” and there is hardly any way to “keep from drowning” [3]. Considering the thousands of life-science databases that have been created [4], that pessimistic view has not become reality, although there are many areas (e.g., NGS) where problems in handling the constantly increasing amounts of data exist. In some areas, international consortia have taken control of data maintenance and management [5].

Scientists can choose from dozens of information retrieval systems [6] to search for data resources. Metadata standards [7] and data-exchange formats have been developed [8], and data warehouse infrastructures have been implemented [9]. For example, the DataCite consortium [10] was founded to support data citation, providing a means to increase the acceptance of research data as legitimate contributions to scholarly records. Those promising technologies only handle the tip of the “data iceberg”, however [11]. The gap between the rate of scholarly publication and the availability of long-term research data preservation remains an open issue [12,13].

A previous publication underpinned this motivation by summarizing the basic requirements for primary data management [14]. In the absence of such management, a scientific staff usually collects a lot of data and then reduces them to a number of theories and conclusions supported by aggregated graphics, tables, or selected materials. Publications contain links that refer to externally managed, supplemental resources where data may be found. Anderson et al. [15] showed, however, that the older an article is, the lower is the chance that the links are still accessible.

### Relationship between data publication and long-term data preservation

**Figure 1:**
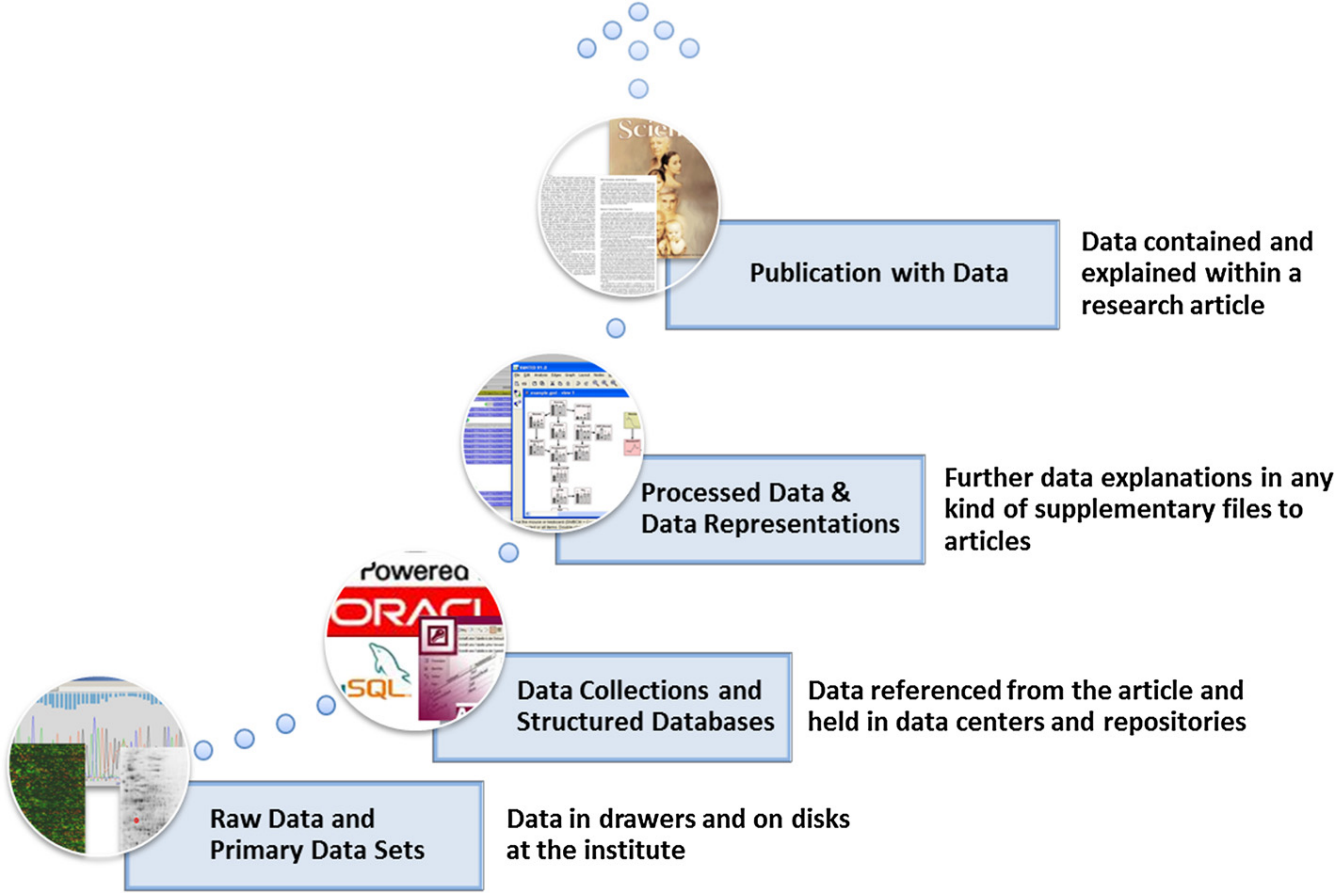
**Publication Process of Research Data Sample.** The data-publication process (inspired by Gray et al. [11]) expresses the different manifestations of research data. At the *top layer* of the process, the journal, author, or scientist takes full responsibility for the publication, including the aggregated data embedded in it and the way the data is presented. For data published in the *second layer*, as supplementary files to articles, the link to the published “Record of Science” remains strong; but it is not always clear at what level the data is curated and preserved and if the criteria for discoverability and re-usability are met. At the *Data Collections and Structured Database layer*, the publication includes a citation and links to the data; but the data resides in and is the responsibility of a separate repository. At the *bottom layer*, most datasets remain unpublished and are consequently not accessible for later reanalysis.

The data life cycle, from experiments to scientific publications, generally follows the schema in Figure 1. Several billion dollars have been invested in the bottom level, the collection of “primary data”. The definition of primary data is not clearly fixed. For some, it is only the raw data from a device, also called “Level 0” data, and for others, it also includes preprocessed raw data without additional analytic processing steps. Still others do not differentiate among the degrees of processing but consider primary data to be all data that is used for scientific publications [16]. Here, we consider primary data to include all of the aforementioned categories. It is important to consider primary data as an investment in order to guarantee its long-term preservation and prevent its being lost, and to ensure that it can be processed in future works including, but not limited to, ad-hoc data-analysis workflows.

Condensed and enriched with metadata, primary data is more valuable than data that is “re-extracted” from articles [17]. This argument is the basis for efforts like the Open Archival Information System (OAIS), which aims to preserve primary data and provide associated information to designated communities [18]. Such comprehensive models are very expensive, however, for short-term and mid-term research projects. One promising alternative is data publication as a separate research result in a “data paper” [19]. In this context, it becomes essential to change the handling and acceptance of primary data within the scientific community. Nelson et al. [20] argued that data publications and data publishers should be honored with a high degree of attention and status.

In general, most scientists are willing to share their primary data, but few actually provide their data to others [12]. Data access is often restricted to project-associated users, or else the original data sets frequently remain in the hands of the scientist who created them. Another issue is the deficiency of metadata annotation. Scientists tend to use personal metadata in the form of remarks or smart file naming if they are not forced to use a standard metadata format by journal or project policies. Technical aspects, such as proper backup and disaster-recovery systems, also affect the prevention of data loss. Besides preserving data files, it is essential to preserve file-access procedures. Here, *data format migration* and *data format emulation* are widely used methods.

### Data sharing

There are already many *public primary data repositories* available free of charge, but they are very domain specific (e.g., the Sequence Read Archive (SRA) [21] and the Gene Expression Omnibus (GEO) [22] for raw NGS-sequencing data and gene expression data, respectively). The common concept is user-controlled data upload and maintenance, but the shortcomings are the dedicated data domains and content. The acceptance rate is quite high, particularly if a journal forces authors to upload the primary data to a repository to get a paper published. The major drawback of the central repositories is clearly the limited number of supported data domains.

Besides the dedicated repositories, free data-sharing platforms and cloud storage (e.g., Dropbox [23] and Google Drive [24]) are becoming very popular as economical alternatives to *project level data infrastructure*. These platforms often have limited security management and do not often support metadata management. More comprehensive data-sharing and publication services are available through data-file archives [25,26]. Examples of well-accepted scientific data repositories are Dryad [27] and figshare.com [28], which not only provide professional support for file sharing, maintenance, and publication by also support persistent identifiers (e.g., DOI and URN). They are, however, centrally hosted by companies that require data-publishing charges, and they do not support tool binding or use as an in-house repository on top of one’s own storage backend.

More flexible and enterprise-sized data repositories that provide long-term, durable access to digital assets are DSpace [29] and Fedora [30]. Both include a variety of preservation and management functionalities as well as support workflows for uploading, approval, and web publication. Fedora is a digital asset-management architecture. Its flexibility carries with it the expectation that users will invest more up-front effort in creating specialized object models and applications. DSpace is aimed to be deployed as an “out-of-the-box” institutional repository (e.g., in Dryad) and supports little to no customization.

There are also some mid-sized and lightweight data repositories like Tranche [31] and CKAN [32]. Tranche is a very flexible repository API for JAVA, and CKAN is a web-based management system for any kind of data. Both can be integrated into existing infrastructures, but they provide no persistent identifiers.

Another alternative is publicly hosted version-tracking systems [33,34]. For an in-house service, there are a number of commercial and open-source systems available (e.g., Subversion [35] and Git [36]). Those are excellent for tracking file histories and preventing accidental deletions, but their support for information retrieval and their facilities for citable data publication are limited. In addition, they are not suited to storing and sharing large binary files, because it is much more difficult to compress and calculate the differences between binary files compared with simple text files, which can strongly influence performance and increase the required storage capacity.

Much more popular and easy to handle, but also very risky, are private file storage, exchange via email attachments, and uploading directly to partners’ in-house file servers. Here, the major problems are unregulated data distribution with the danger of inconsistencies, limited support for persistent identifiers, and potential unauthorized access, data loss, and mess caused by missing metadata. More reliable solutions for data storage and exchange are *Laboratory Information Systems* (LIMS) [37] like LABKey [38] and Nautilus LIMS [39]. In general, LIMS support the case-specific design of interlab and intralab collaborations. Their tight integration with lab processes make them as useful as electronic lab books, but their focus on managing lab processes and their implementation as a closed software system make them difficult to use as a stand-alone, public, citable storage infrastructure.

### Data annotation and citation

The *metadata annotation* of research data is an important prerequisite for the interpretation and reuse of data sets. This is reflected in the manifold metadata schemata that are used in the life sciences. In general, we differentiate between technical and semantic metadata. The latter type has a tight relationship to the particular research domain and comprises its own universe of several hundred metadata schemata. For instance, a systems-biology review summarized 30 standards for metadata and data-exchange formats [40]. Technical metadata covers aspects of the management and processing of digital research resources. Widely used schema for technical metadata are the Dublin Core Metadata Element Set (DCMES) [41] and the closely related DataCite Metadata Schema [42]. The DCMES was developed by scientists and librarians to homogeneously describe digital objects using 15 elements.

Because of the missing metadata-aware file systems, the most popular method of metadata annotation is still the coding into file names and folder structures; and the application of information-retrieval and search-engine technologies is one of the most preferred methods to find relevant data [43]. Desktop search tools like Google Desktop [44] are therefore frequently installed on the scientists’ computers. Furthermore, frameworks like Apache Solr [45] allow embedding a full-text search into data repositories. Such indices are increasingly important in information systems for life sciences, and they are often available in primary data repositories (e.g., the DataCite Metadata Search).

### Data publication

The motivation for publishing research data is not limited to the need to evaluate and appraise scientific results. Data sharing in general is an essential resource for scientific research. Research groups, straddling scientific cooperation and the public, depend crucially on data sharing and public data access. URIs and proprietary database identifiers are frequently used to reference data or supplemental material in manuscripts. Because URIs (i.e., HTTP or FTP URLs) are short-lived in practice, they are not considered to be reliable references. The same is true for proprietary database identifiers (e.g., Genbank accession numbers). Besides such archive-specific identifiers, *long-term resolvable identifiers* are used in the life sciences. Examples are international standardized identifiers such as the Life Sciences Identifier (LSID) [46] and the Digital Object Identifier (DOI) [47]. International initiatives like OAI [48], ELIXIR [49], and DataCite [10] were founded to develop sustainable research networks and infrastructures. Their common aim is to coordinate the development of technologies in conjunction with standards and policies that incorporate the major aspects of data publication: 

1. controlled data formats

2. globally persistent identifiers

3. minimal commonly accepted metadata

### Minimal requirements for primary-data management

The aforementioned challenges in the long-term preservation of primary data and the need for a consistent data-publication process highlight the need for a universal software system for primary-data management and publication. The minimal requirements of such a system are summarized in six tasks:

#### Version management

In order to track the history of data files and associated metadata, an infrastructure for long-term data preservation should support a version-control mechanism. In particular, this affects deletion policy. Primary data is not allowed to be deleted.

#### Metadata

To support future data access, readability, and use, annotation with technical metadata is necessary. Furthermore, metadata is essential for information retrieval, data seeking, and filtering. The intension of technical metadata is to support the automatic processing not featuring semantic interpretation and data analysis.

#### Information retrieval

Because a large number of files is expected, data archives should feature an efficient search function over metadata. This should be supplemented by a text index that supports enhanced search functions and faceted queries.

#### Data publication and citation

In general, primary data cannot be included directly in an article. Therefore, authors add links. In order to make those links stable and resolvable in the long term, support for persistent identifiers that fulfill international standards is essential. This will ensure that digital objects are citable, even if the URL moves or becomes obsolete.

#### Data security

In order to consolidate primary-data management, data sharing, private archiving, and public access should be supported in one platform. This requires permission control, secure authentication, and data-transfer systems.

#### Generality

A system for storing research data has to be generic in order to be universally usable. The system should be sustainable in the sense that it can be maintained and applied across project, infrastructure, and institutional borders. By having a user-friendly design and the capability to be easily integrated into existing workflows and tools, a standardized system should motivate users to publish primary data.

## Implementation

*e!DAL* is an implementation of a lightweight software framework for publishing and sharing research data. *e!DAL* stands for ***e*** *lectronic* ***D*** *ata* ***A*** *rchive* ***L*** *ibrary*. It is a combined open framework and out-of-the-box system for storing, sharing, and publishing research data. The software combines classical file-system concepts with concepts from research-data publication. *e!DAL* is delivered as an API, to be easily usable in existing data frontends, and as a standalone data-repository server. This enables tight integration into existing infrastructures and tools as well as standalone installation as a research-data repository.

### Design and concept

**Figure 2:**
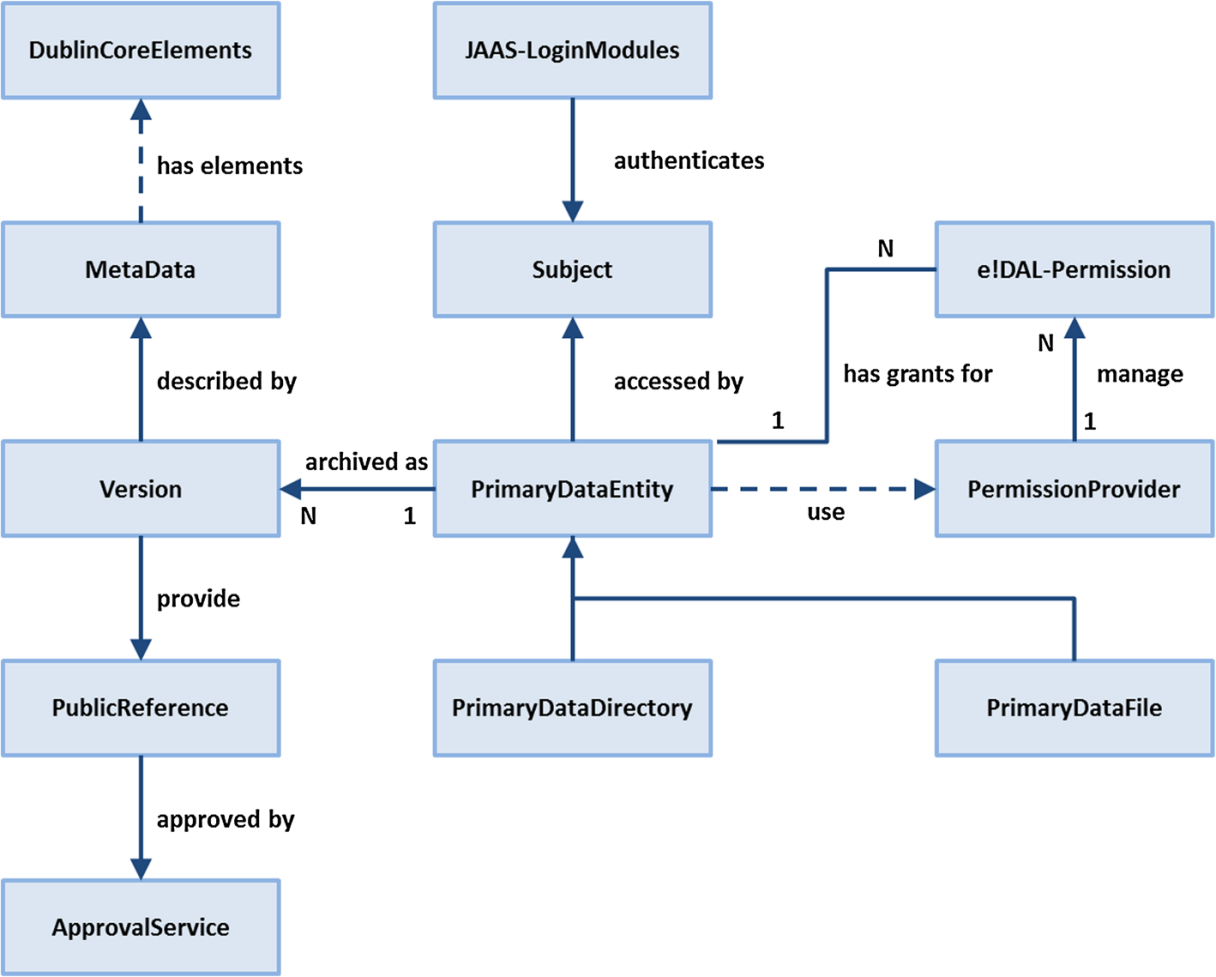
**The *e!DAL * data schema.** Conceptual overview of the major entities of the *e!DAL* infrastructure.

The *e!DAL* core modules and data structure are shown in Figure 2. The object-oriented design makes it possible to keep the implementation scalable and easily maintainable. Twelve JAVA classes implement the core API. Accordingly to the six previously described, minimal requirements for primary-data management, their implementation is described below.

#### Version management

The central element of the data structure is the class PrimaryDataEntity, which is a generalization of the sub-classes directory and file. It provides methods comparable to those of a file system. To implement the data life cycle, all data and metadata updates are recorded as individual versions. When a file is deleted, a last version is created and tagged as “deleted”, representing the end of the version chain. It is not possible to create a new version for the deleted object or to undelete the last version, but all previous versions are still accessible.

#### Metadata

An ISO-accepted minimal set of mandatory technical metadata (e.g., format, creator, and file size) was defined by the Dublin Core metadata standard [41]. *e!DAL* implements this standard and the suitable data types for each element.

#### Information retrieval

Besides support for navigable access to the hierarchically organized data structure, a full text index over the metadata was applied to provide a keyword search, which features fuzzy queries like phonetic or partial string matching. Selected metadata elements as well as the entire set of metadata can be searched.

#### Data publication and citation

**Figure 3:**
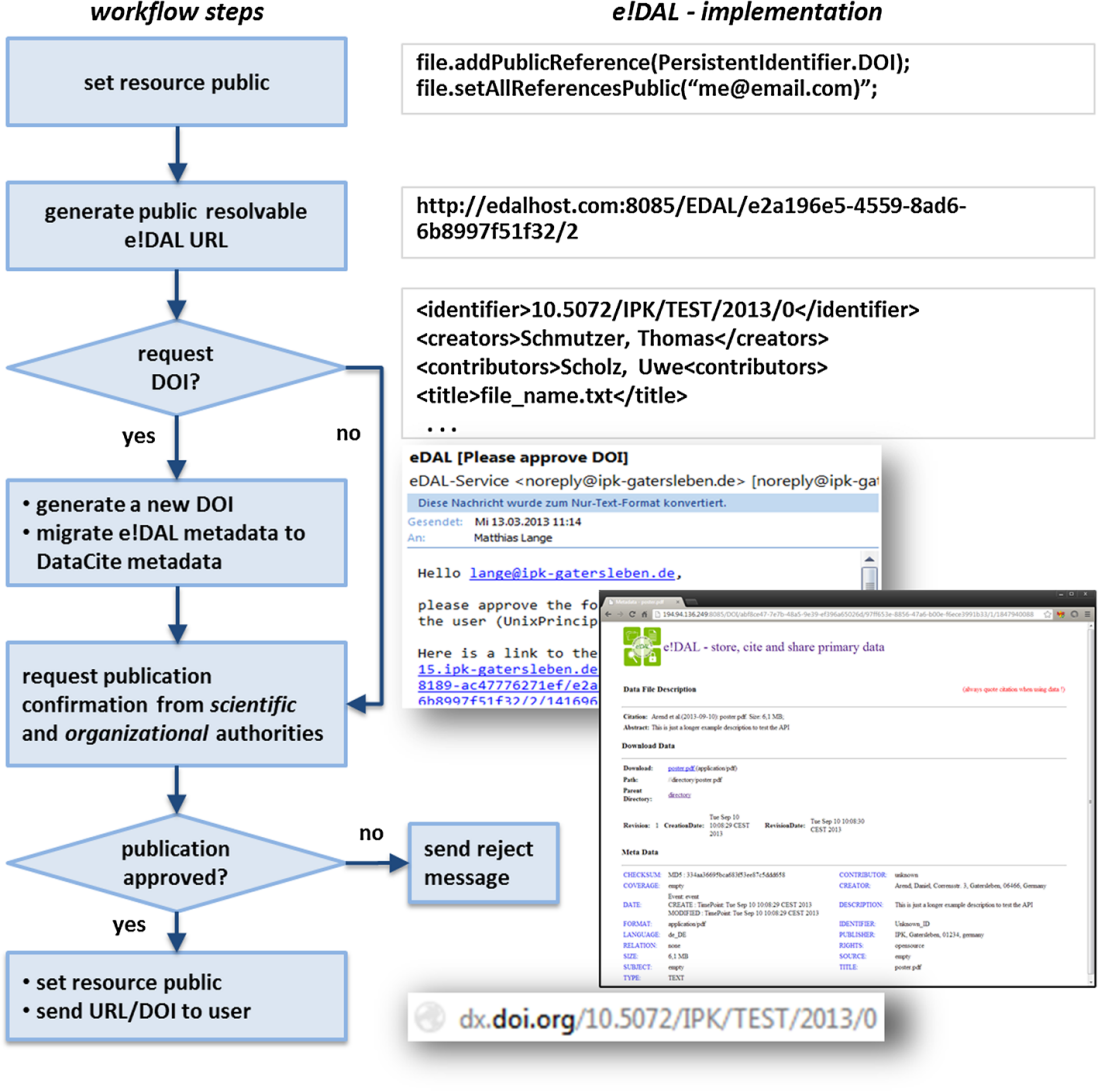
**Data publication workflow.** To ensure a trusted release of research data, a review process has been designed. The first step for a data-publication request is the generation of a “landing page” for the applied citable identifier. The underlying URL is served by the embedded HTTP server. If a dataset has a release date in the future, the page locks the data download. If the user requested a DOI from DataCite, the system generates a unique DOI and migrates the metadata to DataCite-XML format. After the reviewer approves the publication, the DOI request is sent to the DataCite REST web service. Finally, the user gets an email notification with the accepted DOI or URL.

The PublicReference component binds to services, which provide persistent identifiers. Because the identifiers represent scientific publications, the assignments must be permitted by the responsible parties. Therefore, *e!DAL* provides a flexible approval workflow, illustrated in Figure 3, and allows the evaluation of data publication requests by a hierarchy of reviewer decisions. Each request triggers a notification for the reviewers, who then exclusively or collaboratively decide to permit or reject it. This system is based on an asynchronous email notification system with reminder and default decisions for unresponsive reviewers.

#### Data security

*e!DAL* features a fine-grained access control to sensible API methods^a^, which is monitored by the JAVA Authentication and Authorization Service (JAAS) [50]. The concept is that the executing code (thread) is owned by a particular subject (i.e., a user or group), which is assigned by an authentication process when a connection to *e!DAL* is requested. The JAVA-embedded JAAS authentication module enables the transparent use of standard authentication services (e.g., Kerberos, Unix, and Microsoft Windows logins) as well as customized user management.

#### Generality

*e!DAL* supports stand-alone and client-server architectures. It can be integrated into JAVA applications as an embedded local archive or as a central archiving system. With support for JAVA Remote Method Invocation (RMI) [51], several application-specific or even project-specific central repositories can be easily operated.

### Technology

**Figure 4:**
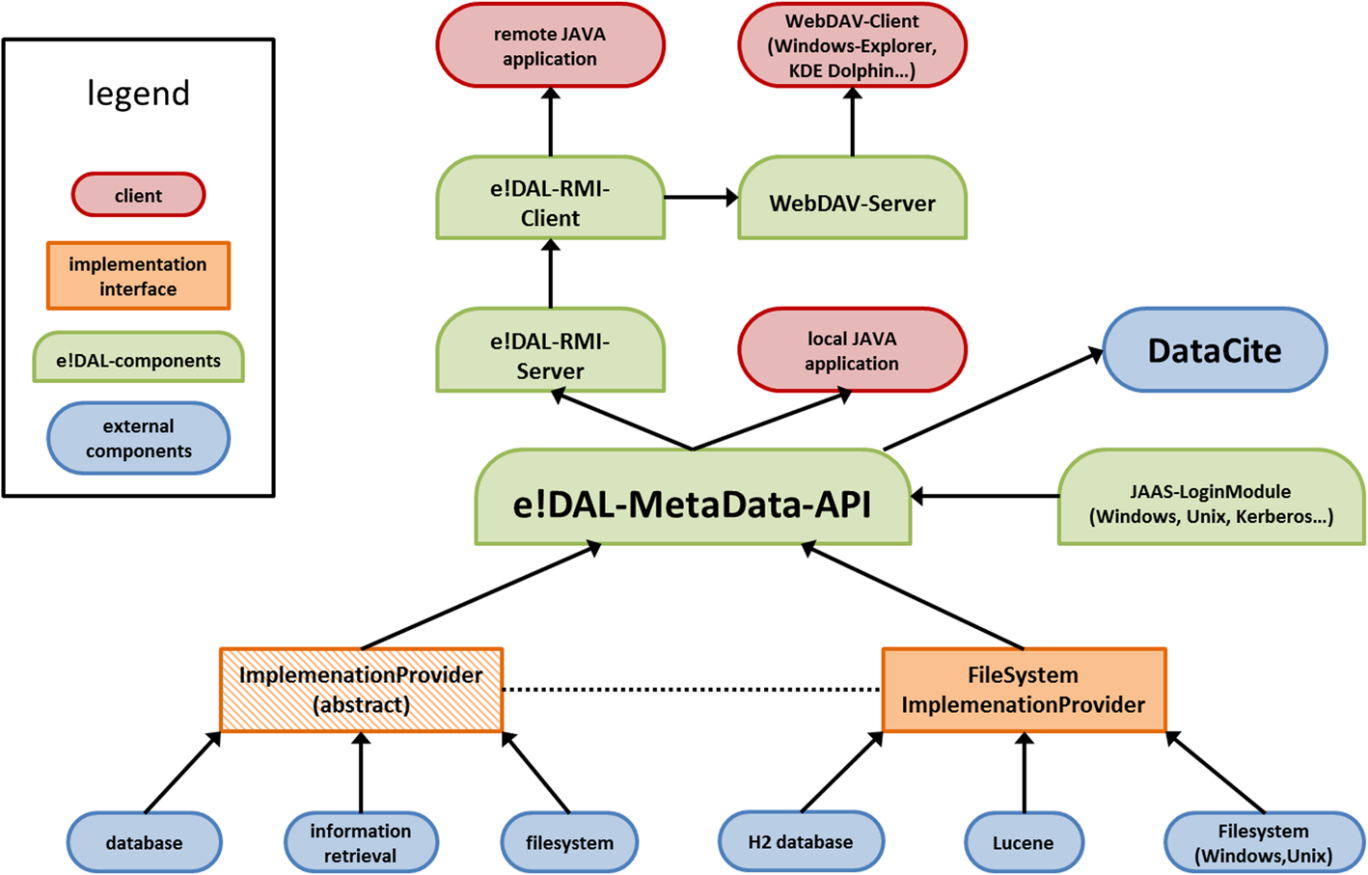
**Architecture of *e!DAL *-API.** The green nodes are the parts of the core *e!DAL*-API, the *e!DAL*-server, and the *e!DAL*-client packages. The yellow nodes represent the implementation interface, and the blue nodes represent the backend components. The red nodes symbolize possible applications.

The system architecture of the *e!DAL* implementation is shown in Figure 4. The persistence of the data structure is implemented by the H2 [52] relational database management system with Hibernate [53] as an object-relational mapper and Ehcache [54] as an offloading database cache. This storage-layer implementation holds all the technical metadata, permissions, version information, and publication metadata. Apache Lucene [55] is applied as an implementation of the information-retrieval interface, integrated on top of the Hibernate persistence layer by the Hibernate Search package [56].

The data-byte stream is stored as a file in a mounted file system. In order reduce file-system load, data files are grouped into chunks, which are distributed into a self-tuning directory structure. Another feature of the storage backend implementation is easy maintainability (i.e., copy, clone, and backup). Thus, it is sufficient for the administrator to use file-system backup and maintenance tools. The registration and assignment of citable persistent identifiers to data entities (files or directories) is implemented by a generic interface, which includes the data publication-service connectors. The flexible interface supports an extensible number of data citation services. Currently, the reference implementation supports the generation of URLs and DOIs. The latter are provided by the DataCite Consortium [10] and handled by the *e!DAL*-DataCite connector. For a particular data object, the interface: 

1. migrates the *e!DAL* metadata to the metadata format of DataCite,

2. generates a landing page in the *e!DAL* HTTP module,

3. assigns the DOI to the *e!DAL* URL, and

4. submits all information as XML to the DataCite web service.Before the DOI-registration workflow is executed, the approval process checks whether the publication is allowed (see Figure 3). In the implementation, three reviewers are configured by registering their email addresses as either a legal, scientific, or master reviewer with veto permissions. For each publication request, emails are sent with “accept” and “reject” links. After the DOI or URL assignment has been accepted or rejected, the user is informed by email.

JAAS is used to implement the API-level permission-control system, enabling each session to run in a dedicated user context where each relevant security method is encapsulated into an authorization check. A list of user method-permission rules is persisted in the H2 database backend. As a default, the user who created an object is the object’s owner and gets all permissions. Directory permissions are inherited by all newly created files. Further user permissions can be granted by the object owner or by users who have the right to set permissions. Permissions can be set for individual users and also for user groups or different users roles.

The user runtime context is set by calling the login method in a JAAS-supported login module. Three default modules are available: native Windows and UNIX logins as well as the LDAP Kerberos authentication service. Further customized login modules can be added. A template is provided in the JAVA class SampleLoginModule. To minimize the effort required to check the permissions of dozens of API methods and to make the code robust, we applied aspect-oriented programming using the AspectJ framework [57]. The permission code is defined once as a security aspect and weaved into the source code for every public API method.

## Results and discussion

*e!DAL* offers a flexible and efficient lightweight framework that transparently incorporates features for research data management, such as storage, data sharing, and data citation, into existing tools and storage infrastructures. We released a multi-module system that can act as: 

• a direct-linked API for a local non-shared storage,

• a remote API to enable applications transparent access to distributed data,

• an out-of-the-box data repository server for hosting shareable data repository, and

• a remote file system with enhanced features for metadata and version control.

### Features

#### The JAVA API

To feature JAVA applications with *e!DAL*, the API^b^ needs to be imported into the JAVA source. For an automatic import of the suitable dependencies, it is available as a Maven [58] artifact^c^ and mirrored in the Maven Central repositories. For manual dependency management, fat JAR archives are available for download.

The *e!DAL* API is designed like the JDK file-system API with additional classes and methods for versioning, metadata annotation, information retrieval, and data publication. Like an ordinary file system, the *e!DAL* backend needs to be mounted and parameterized with: 

1. storage-backend implementation (see Section Technology),

2. the JAAS-authenticated login context, and

3. parameters for the data-publication process (i.e., reviewer email addresses, SMTP server, or an optional HTTP(S) proxy).

#### Standalone data-repository server

In order to support shared and collaborative access, *e!DAL* has a server module, which provides an RMI service to handle native JAVA clients, a WebDAV server to offer access as a network file system, and an HTTP server to support access by any web browser. According to the client capabilities, the supported *e!DAL* features range from browsing and downloading published data (HTTP), to providing a metadata-aware and version-aware remote file system (WebDAV), to providing full-featured API access (RMI). This wide range of functionality has been implemented to support application scenarios and desktop users that need data access in a file browser. The implemented WebDAV interface [59] allows users to mount a connection to *e!DAL* repositories as a network drive. Desktop tools, such as Windows Explorer, Linux KDE Dolphin, or any other WebDAV-compatible browser, are supported.

WebDAV provides functionality for versioning and metadata, but its visualization and handling are commonly not implemented in file browsers. This is overcome by virtual folders and files. In doing so, an *e!DAL* file is mapped as a virtual WebDAV folder, which includes the files of all versions. Each version is linked to a virtual XML file, which makes it possible to inspect and edit metadata.

### Software quality

Primary data is a major research asset, and any data-managing software must fulfill basic quality criteria. Hence, software quality was a major aspect while developing *e!DAL*.

#### Development, scalability and code-quality control

Besides platform independence, the major advantage to implementing *e!DAL* in JAVA is the availability of open-source standard frameworks, like authentication services (JAAS), persistence frameworks (Hibernate), code-weaving tools (AspectJ), and build-management and dependency-management systems (Maven). Furthermore, JAVA is widely used in bioinformatics to develop graphical user interfaces, data management infrastructures, and web applications.

Following lessons learned in the past decade of active software development in research projects, published studies [60], and guidelines for agile software development [61] and consequent automated testing (using JUnit), we focused our development on the core code and performance optimization. On the one hand, this guaranteed the use of modern, best-performing frameworks, and on the other hand, it prevented us from wasting time on developing proprietary, error-prone code for components such as security, database connection, and user management.

#### Performance benchmarking

**Figure 5:**
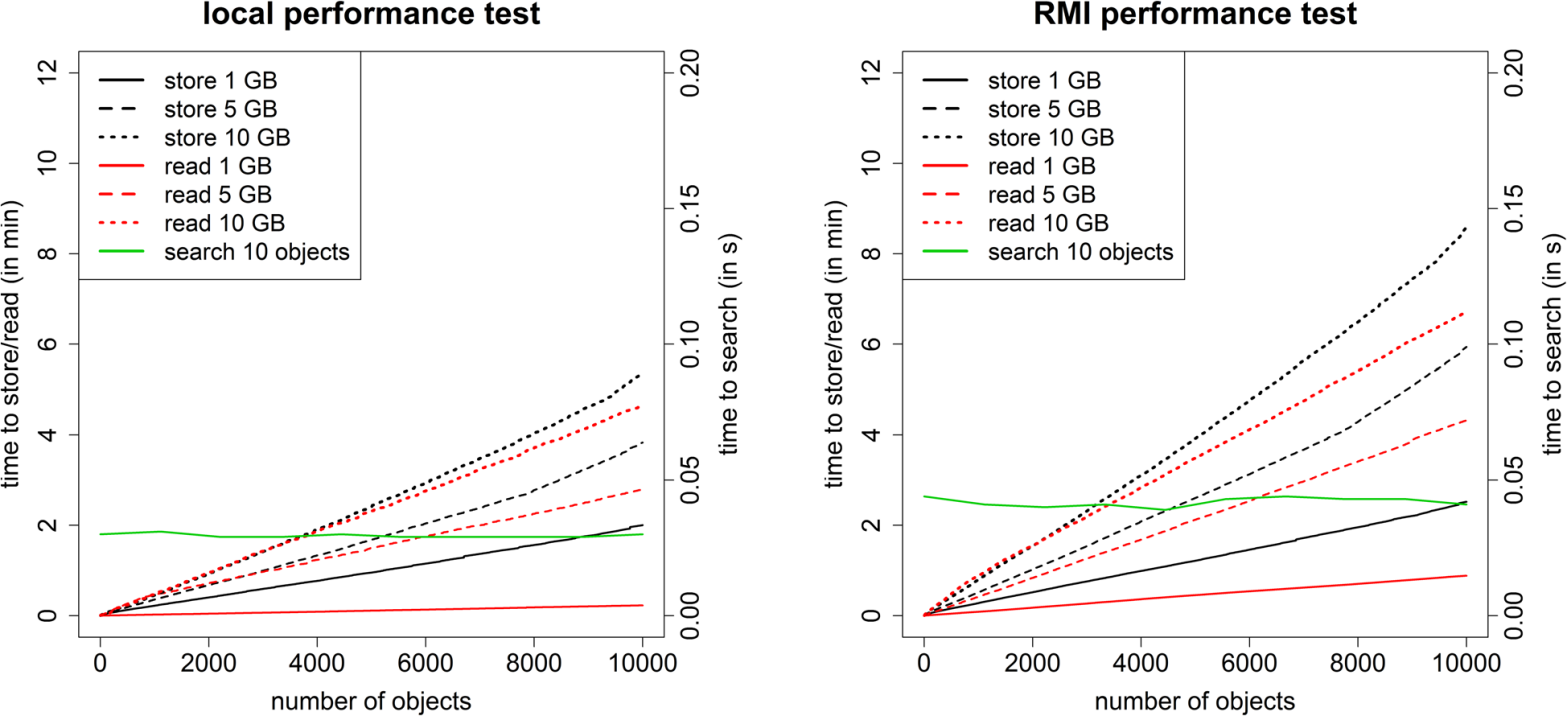
**Performance benchmark.** Performance tests for local embedded (left) and server-client architecture (right). We used data sets with 10,000 files in 100 folders, but with different file sizes (0.1, 0.5, and 1.0 MB). The left y-axis shows the time required to store all of the objects and read them again to a new directory. The right y-axis shows the performance of the index-based search. Using the read/store test set, we sent queries, which gave exactly 10 results each. All tests were executed on a Linux system with a six-core AMD Phenom II X6 1055T Processor at 2.8 GHz and 64 MB heap space for the JAVA virtual machine. The system had a 1-GB ethernet connection and a SATA hard disk (7200 Rpm).

We ran several performance tests to evaluate the efficiency of the example implementation. Here, we explain two tests and compare the results of *e!DAL* with those of a native file system. The benchmarks show the performance of *e!DAL* while storing and reading objects and while searching for a fixed number of objects, depending on the number of stored objects. All results are summarized in Figure 5. All tests were executed with a local embedded *e!DAL* system as well as with the server-client architecture.

In the first test, the time the API needed to store and read 10,000 objects was nearly linear and depended only on the file size, because the read and write operations of the file system needed more time. In general, the read function is faster than the write function, as there are fewer database operations necessary. Furthermore, it is clear that all the tests over the server-client architecture needed a bit more time, because all the data had to be serialized and transferred over a network connection. While supporting more features than an ordinary file system, finished the storage of 10,000 files, each about 0.1 MB in size, in about 2 minutes, which is close to the performance of a Windows NTFS or Linux EXT4 file system. The performance in client-server mode was comparable to that of network-based file transfer protocols such as CIFS or NFS.

The second test showed the performance of the *e!DAL* search functions, which clearly outperformed those of local or network file systems. As expected, the performance was independent of the number of stored objects, thanks to the use of the text index. The small differences between the values for the local tests and the tests over the server-client architecture were caused by fluctuations in the network latency.

### Use cases

Although *e!DAL* is a generic framework for research-data management, some specific use cases demonstrate its manifold applicability.

#### GUI support and demo

**Figure 6:**
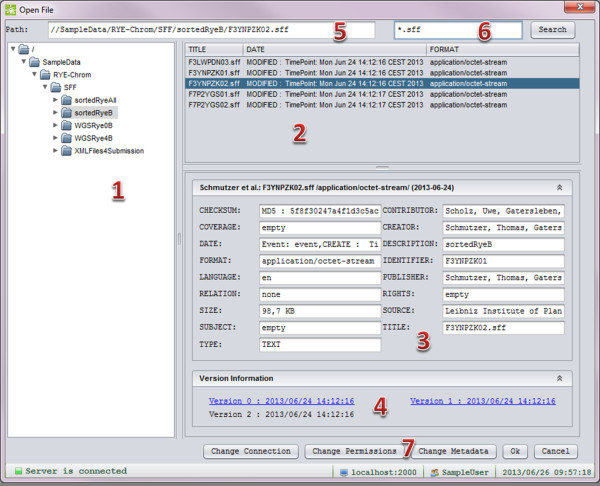
**EdalFileChooser dialog.** The eDAL-FileChooser dialog comprises several components as follows: (1) a file tree to navigate through the stored directories, (2) a window to display all files and subdirectories in the chosen folder, (3) textfields to display the meta information of the chosen version (to change the meta information, the user has to double click on a textfield), (4) a table to show all stored versions of a digital object (the user has to switch between the versions by marking a field), (5) a textfield to show the complete path of the current object, (6) a textfield for search function, and (7) open dialogs to change permissions or metadata.

In order to support seamless integration into graphical JAVA user interfaces, a file-chooser dialog was implemented, with functionality and handling like the default file open/save dialog, but with enhanced features. As illustrated in Figure 6, it behaves like a basic file explorer, allowing users to browse and select *e!DAL* objects, edit metadata, and manage permissions for stored objects, and it features a search box to use *e!DAL*’s information-retrieval functionality.

For the purpose of demonstrating *e!DAL*’s features and supporting a quick-start guide for the API, a multipurpose “*e!DAL*-Installer” Webstart application is available at the project website. The initial wizard panel makes it possible to either install an *e!DAL* server and generate startup scripts or run through a demo, allowing users to test the different modules and functions like the EdalFileChooser dialog or the publication process. The e!DAL demo includes an NGS dataset, giving an impression of the importance of metadata handling. e!DAL uses the Dublin core to provide metadata for stored files. That allows users not only to provide information such as the title or the author but also to provide useful information like taxonomic identifiers or file descriptions that help users to find data. An example for an NGS metadata set is given in Table 1.

**Table 1:** **Metadata of an *e!DAL * managed Next Generation Sequencing test data set of shortened sequence files (DOI: 10.5072/IPK/Test/2013/0)**

**Metadata element**	**Value**
Contributor	Scholz, Uwe, IPK Gatersleben, Corrensstr.3 OT Gatersleben, 06466 Seeland, Germany
Coverage	Sequences of pooled flow sorted rye A chromosomes (1R-7R)
Creator	Schmutzer, Thomas, IPK Gatersleben, Corrensstr.3 OT Gatersleben, 06466 Seeland, Germany
Description	Individual rye chromosomes were isolated by flow sorting and shotgun sequenced by 454 Titanium pyrosequencing
Identifier	ERS077775
Publisher	Leibniz Institute of Plant Genetics and Crop Plant Research Gatersleben
Rights	http://http: //www.ebi.ac.uk/about/terms-of-use
Size	98.7 KB
Source	Secale cereale L.; CoL tax ID 9793059
Subject	NGS, Rye
Title	F32CY7K01.sff

#### Systems biology

To enable the community-based development and sharing of biological models, a sustainable data-sharing infrastructure must be provided. Several approaches exist. For example, BioModels [62] reflects the need for an integrated repository of curated models. That approach does not, however, support community-based editing and versioning. The COMBINE archive [63] is a data format for storing models, associated data, and procedures, but it does not provide a storage infrastructure for collaborative data sharing or publication. WikiPathways [64] is a collaborative platform for curating biological pathways, but it focuses on graphical pathway representation and not on SBML [65] models.

To address those shortcomings, we applied *e!DAL* as a repository for systems biology data that supports collaborative editing and access to models among different partners. In doing so, we developed a plug-in for the systems biology framework VANTED [66]. The ordinary SBML file-storage dialog has been enhanced towards being an extended model-sharing infrastructure. The user has the option to select a local or shared e!DAL repository. Version control, technical metadata, as well as a within-model searches are supported.

#### DOI resolving and HTTP(S) server

Finally, data can be published as URLs using the embedded HTTP(S) service or as DOIs using the DataCite infrastructure. The embedded HTTP(S) server provides direct access to published *e!DAL* objects via a browser by rendering landing pages that comprise all relevant metadata and download links. To get an impression of the service and how it looks, we set up a test instance of the API over a secured web server and provided some of the NGS data sets that we used for the demo application. The dataset is available under the DOI: **10.5072/IPK/Test/2013/0**, which can be directly accessed over the DOI resolver (http://dx.doi.org).

### Delimitation to existing software

In Table 2, a comparison to a popular primary data-management system is given. The focus of *e!DAL* was to meet a set of minimal requirements for primary-data management but not to create a comprehensive repository or information system. This principle enables *e!DAL* to serve as a component of full-feature data repositories or as an embedded module in single tools. In summary, the major benefits of *e!DAL* compared with existing systems are its lightweight embeddable design, customizable data publication and DOI registration workflow, and the seamless integration of data storage, sharing, and publication in one thin, reusable API.

**Table 2:** **Comparison *e!DAL * with selected primary data management system**

	**Persistent**	**Generality**	**Metadata**	**Information**	**Data publication &**	**Data**	**Version**
	**identifier**			**retrieval**	**citation**	**security**	**managment**
e!DAL	✓	✓	✓	✓	✓	✓	✓
SRA [21]			✓	✓	✓	(✓)	(✓)
GEO [22]			✓	✓	✓	(✓)	(✓)
CKAN [32]		(✓)	✓	✓	✓	(✓)	✓
Tranche [31]		✓	✓	✓		(✓)	✓
Figshare [28]	✓	(✓)	✓	✓	✓		
DSpace [29] / Dryad [27]	✓		✓	✓	✓		✓
Fedora [30]		(✓)	✓	✓	(✓)	✓	✓
SVN [35]		(✓)		(✓)		✓	✓
GIT [36]		(✓)		(✓)		✓	✓
Google Drive [24]		(✓)		✓	(✓)	✓	✓
Dropbox [23]		(✓)		✓	(✓)	✓	✓
Nautilus LIMS [39]		(✓)	✓	✓		(✓)	✓
LABKey [38]		(✓)	✓	✓		(✓)	✓

This overview summarize the implementation of the minimal requirements for primary data management in those systems that are mentioned in section Data sharing. When a system fullfill a requirement completely, it is marked by ✓. If basic features are implemented the field is marked by (✓). If no concrete information is available or the requirement is not met, the field is left blank.

### Outlook

#### Planned future development of new features

Currently, *e!DAL* has limited support for remote API access for programming languages other than JAVA. In the current release, only JAVA-RMI is supported. The extension towards a RESTful API is planned for the next release. The support of this platform-independent protocol will enable direct access to the *e!DAL*-API for a wide spectrum of programing languages and infrastructures. Another future *e!DAL* extension will be the use of scalable, distributed architectures, such as JAVA clustering solutions, and distributed file systems like Apache HDFS [67]. For this, the next release will virtualize the backend file-system access. One promising framework is the Apache Commons Virtual File System [68]. Using the high number of available drivers, e!DAL will be able to support cloud storage and distributed file systems as well as local file systems as the storage backend.

#### Hosting data repositories

Besides e!DAL’s application to sharing systems biology data, the aim is to use the e!DAL technology platform as a host for data repositories. An example is its application in the German Plant Phenotyping Network. The consortium is developing novel concepts and technologies in the field of high-throughput plant phenotyping. One task is to find a standardized exchange format that comprises primary data, analysis results, and technical and semantic descriptions. One promising solution is the ISA-TAB [69] format, which is a general format for the annotation of experiments that supports several ontologies. *e!DAL* is planned for use as a repository for published ISA-TAB formatted DPPN experiments. A first data set was published under the DOI http://dx.doi.org/10.5447/DPPN/2014/0. More *e!DAL*-hosted data repositories are underway and will be announced at the *e!DAL* project web site.

## Conclusion

*e!DAL* is a lightweight software framework for the management, publication, and sharing of research data. It is designed to turn sets of primary data into citable data publications. This is particularly important for the life sciences, where there is a big gap between the rate of data collection and the rate of data publication. *e!DAL* is available as a local API, a remote file system, or a server for individual data repositories. Its well-defined API supports seamless integration into existing data-management software and infrastructures. In addition, *e!DAL* can be used as a supplement to manage primary data; for instance, the examples presented showed its application to systems biology, genomics, and phenotyping. Furthermore, *e!DAL*’s modular architecture and incorporated standards ensure version management, documentation, information retrieval, persistent identification, data security, and data publication. Developed within a context for the life sciences, e!DAL has many generic features that make it easily and readily applicable to other areas of science faced with similar needs.

## Availability and requirements

• **Project name:***e!DAL*

• **Project home page:**http://edal.ipk-gatersleben.de

• **Operating system(s):** Platform independent

• **Programming language:** JAVA

• **Other requirements:** JAVA 1.7 or higher

• **License:** GNU General Public License (GPL) v2

• **Any restrictions to use by non-academics:** none

## Endnotes

^a^ Sensible API methods are those related to actions for *creation*, *modification*, *read*, *store*, *metadata change*, *permission change*, and *publication*. Details are listed in the documentation for the JAVA class GrantableMethods.

^b^ For the most recent versions and documentation, please refer to http://edal.ipk-gatersleben.de/.

^c^ The Maven artifacts for *e!DAL* are published in Maven Central with the group id de.ipk-gatersleben. A template for project integration is available at http://edal.ipk-gatersleben.de/download/maven.html.

## Competing interests

The authors declare that they have no competing interests.

## Authors’ contributions

ML initiated the project. DA, ML, SF, and CC designed the data structure and tested the software. DA and ML implemented the core API. DA implemented the server and client API as well as the demo showcase, HTTP(S) handler, and installer. DH designed the approval workflow for the data publication. JC implemented the EdalFileChooser GUI. ML and DA drafted the manuscript. US supplied the use cases and NGS test data sets and supervised the project. All authors read and approved the final manuscript.
